# The suitability of smartphone camera sensors for detecting radiation

**DOI:** 10.1038/s41598-021-92195-y

**Published:** 2021-06-16

**Authors:** Yehia H. Johary, Jamie Trapp, Ali Aamry, Hussin Aamri, N. Tamam, A. Sulieman

**Affiliations:** 1 Medical Physics Department, General Directorate of Health Affairs in Aseer Region, Abha, Saudi Arabia; 2 Science and Engineering Faculty, Queensland University of Technology, Brisbane, Australia; 3 Nuclear Medicine Department, King Saud Medical City, Riyadh, Saudi Arabia; 4 Medical Physics Department, King Saud University Medical City (KSUMC), Riyadh, Saudi Arabia; 5 Physics Department, College of Sciences, Princess Nourah Bint Abdulrahman University, P.O Box 84428, Riyadh, 11671 Saudi Arabia; 6 Radiology and Medical Imaging Department, College of Applied Medical Sciences, Prince Sattam Bin Abdulaziz University, P.O.Box 422, Alkharj, 11942 Saudi Arabia

**Keywords:** Biophysics, Natural hazards, Physics

## Abstract

The advanced image sensors installed on now-ubiquitous smartphones can be used to detect ionising radiation in addition to visible light. Radiation incidents on a smartphone camera’s Complementary Metal Oxide Semiconductor (CMOS) sensor creates a signal which can be isolated from a visible light signal to turn the smartphone into a radiation detector. This work aims to report a detailed investigation of a well-reviewed smartphone application for radiation dosimetry that is available for popular smartphone devices under a calibration protocol that is typically used for the commercial calibration of radiation detectors. The iPhone 6s smartphone, which has a CMOS camera sensor, was used in this study. Black tape was utilized to block visible light. The Radioactivity counter app developed by Rolf-Dieter Klein and available on Apple’s App Store was installed on the device and tested using a calibrated radioactive source, calibration concrete pads with a range of known concentrations of radioactive elements, and in direct sunlight. The smartphone CMOS sensor is sensitive to radiation doses as low as 10 µGy/h, with a linear dose response and an angular dependence. The RadioactivityCounter app is limited in that it requires 4–10 min to offer a stable measurement. The precision of the measurement is also affected by heat and a smartphone’s battery level. Although the smartphone is not as accurate as a conventional detector, it is useful enough to detect radiation before the radiation reaches hazardous levels. It can also be used for personal dose assessments and as an alarm for the presence of high radiation levels.

## Introduction

Human beings are exposed to ionizing radiation from human-made and natural sources (terrestrial, cosmic, and internal radiation). The annual dose from background radiation differs from one place to another in the earth’s crust. Nowadays, the increase of ionizing radiation in medicine (imaging and radiotherapy) and industrial activities and weapon increase public attention due to its possible effects. On average, general population receive 2.4 mSv (75%) and 0.6 mSv (25%) from natural and artificial exposure^1–3^. The annual amount of background radiation has wide variability from about 2.0 to 8.0 mSv/year, depending on the altitude, location of the earth’s surface, and human practices and activities^4^. Public awareness of ionizing radiation and its risks has increased dramatically since the Fukushima accidents in the matter of the radiation risk from nuclear power plants and the release of radioactive materials^5^.

Radiation can be used in various applications, and approximately 23 million workers are occupationally exposed to ionizing radiation worldwide^6^. Workers in medical imaging, security, environmental monitoring, and other fields need to be protected from high doses of ionizing radiation. Practical and easily accessible dose monitoring is therefore crucial to ensure the safety of workers and the public. Radiation dose measurements in hospitals and industrial settings have traditionally used Geiger–Muller counters as an alarming method due to its ability to amplify the signal. However, it cannot be used as a personal dosimeter because the signal is independent of the incident radiation that created it^7^. Proper personal monitoring devices should have the ability to detect and register accumulated incident radiation for a specified period.

Similarly, instruments that use a combination of scintillation and semiconductor detectors can provide excellent detection efficiency for high energy gamma rays, including information about incident energy. Though small scintillation detectors can be manufactured while maintaining excellent performance, they are quite expensive, and their output is difficult for a general audience to analyze. The Complementary Metal Oxide Semiconductor (CMOS) image sensors used in smartphone cameras, on the other hand, can detect ionizing radiation photons such as X-rays and high-energy gamma rays^8^. The photodiodes in the core of each CMOS pixel are designed to detect visible light photons, but they are also sensitive to X-ray and gamma radiation^7^. Dominant visible light signals can be easily blocked using a layer of black tape to cover a camera’s lenses. The signals created in the CMOS pixels are then a measure of the amount of X-ray and gamma photons hitting the camera^9^.

Smartphones that have CMOS cameras have been widely adopted throughout the world, which makes them potentially useful as tools for monitoring radiation in cases of civil emergencies such as the Fukushima disaster, or more mundane situations such as radiation exposure while traveling in an aircraft. With the drastic increase of nuclear energy and theranostic nuclear medicine application, there is a need to develop a technique to use the available smartphone in triage incidents or use it as an alarm by non-professional personnel. A range of smartphone applications has been developed that claim to measure ionizing radiation dose rates through the CMOS sensors on board. Modern smartphone cameras have advanced features, such as accelerated camera pixel intensity, higher image quality, and greater rapidity. These features allow smartphones to be especially useful in detecting radiation^7^.

The usability of smartphones as dose alerts has been investigated using an android app called RadioactivityCounter^10^. The WikiSensor app, available for iPhones for less than a dollar, also can detect radioactivity^11^. To use smartphones as radiation alarms, the radiation responses of smartphones should be characterized carefully. This work aims to report a detailed investigation of a well-reviewed smartphone application for radiation dosimetry that is available for popular smartphone devices under a calibration protocol that is typically used for the commercial calibration of radiation detectors.

## Materials and methods

### RadioactivityCounter app

**Figure 1:**
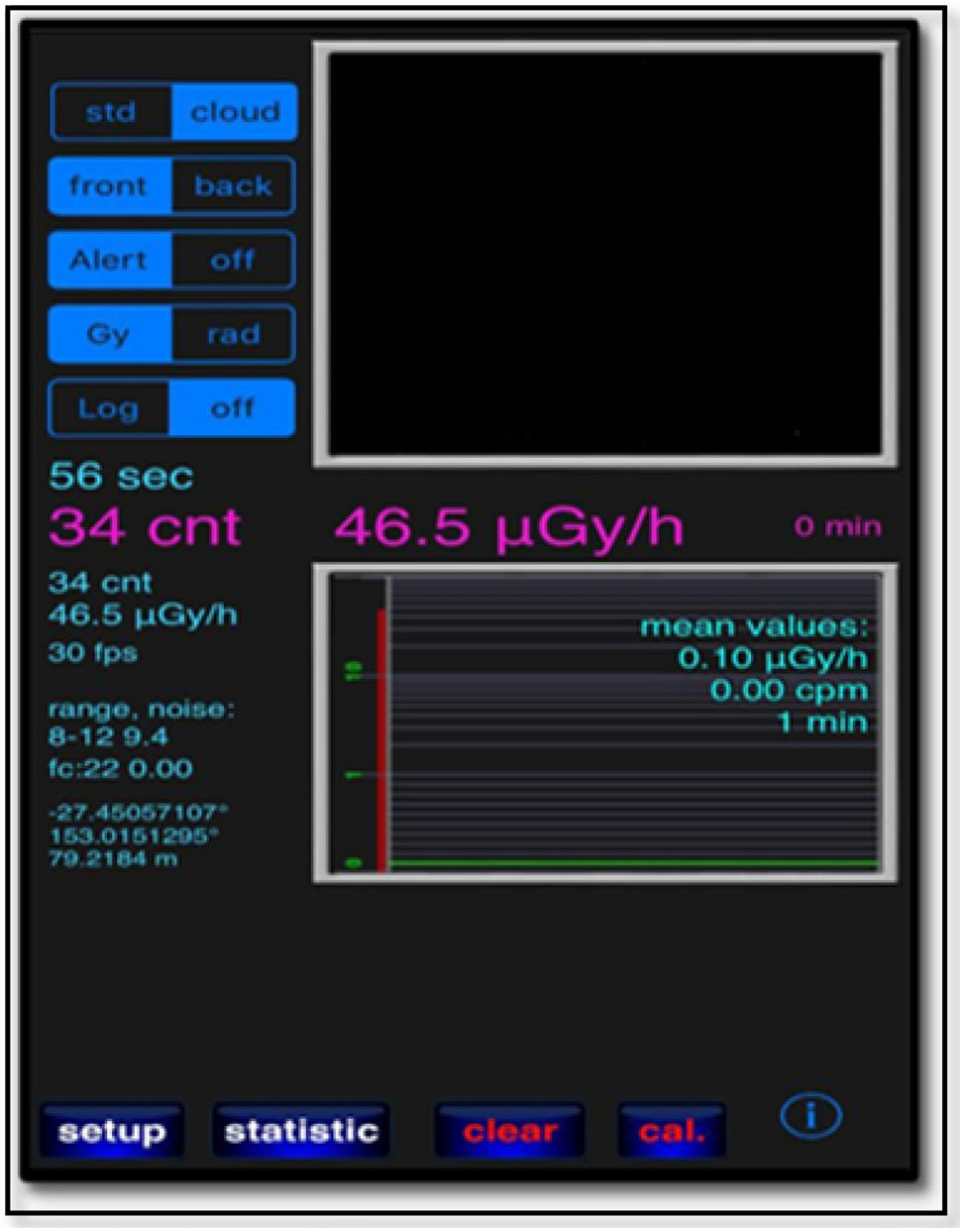
Screenshot of the RadioactivityCounter app, including the camera (front or back can be used), alarm panel, measurement scale (µGy/h), log button (for recorded data to be saved), setup button (to adjust background app activity) and statistic button (to display data as a spectrum or as a bar graph that can be converted to a Comma Separated Values (CSV) file and sent by email).

The RadioactivityCounter app installed on an iPhone 6S was tested using front and back cameras for the photons dose rate in the range of µGy/h. The data is updated every minute automatically and saved. A screenshot of the app is shown in Fig. 1. The app measures the detected radiation in microgray per period, and is well-documented on the developer’s website (www.hotray-info.de). RadioactivityCounter processes data from a camera’s CMOS sensor and records the frequency and number of times it interacts with energetic particles or photons over a specified period. The signal is converted into the dose received by the sensor. RadioactivityCounter was chosen for this study, as it has been reported in the literature to have the most sophisticated calibration process among the apps that have been tested^12^. When launching the application after fully covering the camera lens by black tape, the CMOS sensor’s noise level can be determined through a rapid automatic procedure. During the first use of the application, the background radiation level can also be measured. The default calibration factors are specific to a wide range of different devices. Furthermore, the default values for the background radiation levels and the calibration factors can be adjusted in light of more accurate data. Once a measurement is taken in the desired time frame, the data is automatically recorded, saved, and sent to an email address.

### Measurements with calibrated ^137^CS source

#### Dose rate dependence

**Figure 2:**
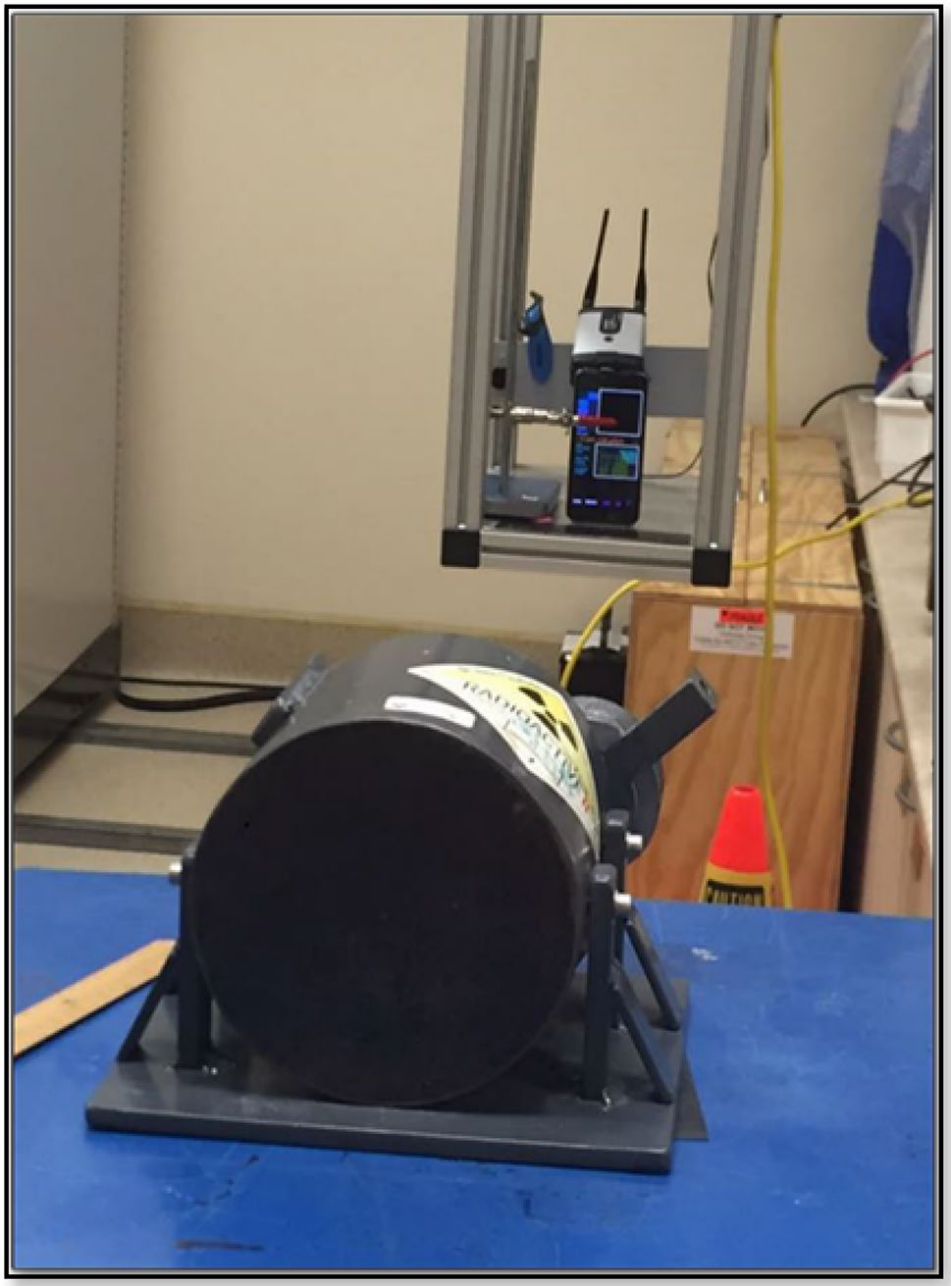
Irradiation and measurement setup.

To evaluate the dose rate dependence of the CMOS response, the RadioactivityCounter app was calibrated using a specific source (^137^Cs 661.6 keV gamma emitter) with serial number 3702GF, traceable to the Australian Government’s primary authority on radiation protection and nuclear safety (ARPANSA). The front and back camera lenses of the smartphone were covered with black tape with a small sheet of Aluminium between the tape layers; however, only the front camera was tested as it is reported to be more sensitive^11^. The camera sensor noise calibration routine ran automatically for four minutes after the app launched, and the sensitivity of the CMOS began at around ten µGy/h^11^. The smartphone was located on a suitable movable platform in front of the calibration source (Fig. 2). The radioactive source (^137^Cs) was located inside a shielding block to prevent radiation leakage. The distance between the source and the phone was varied to attain different dose rates. The app recorded the mean values of the measured data automatically every minute. Each measurement point was a three acquisition which was acquired over three minutes to ensure that the signal had sufficient time to stabilize. The distance variations were performed using a remotely controlled programmable gantry. The minimum distance was 30 cm since a shorter distance can lead to collisions between the source and the gantry. The other distances (in cm) tested were 50, 75, 100, 125, 150, 175, 200, 250, and 300. Data in the app (Counts per minute (CPM), µSv/h dose, time and mean CPM) was automatically recorded every minute, stored, and then sent to the default email address in the form of a Comma Separated Values (CSV) file. Finally, all of the data obtained was analyzed and presented using Microsoft Excel.

#### Angular dependence tests

The smartphone was tested by exposing it to the ^137^Cs source that was 50 cm away, with an expected dose rate of 37.3 µSv/h. The phone was then rotated at different angles (0°, 30°, 45°, 60°, 90°, 135°, and 180°), as shown in Fig. 2. The acquisition time for each measurement was also 3 min for each specific angle. Data in the app (CPM, µSv/h dose, time, and mean CPM) was automatically recorded every minute, stored, and sent to the default email address in the form of a CSV file. Finally, all of the acquired data was analyzed and presented using Microsoft Excel.

#### Measurements at radioactive pads

Field measurements were taken on calibration pads located at the Queensland Health Forensic and Scientific Services facility. The five pads are made of concrete and contain different concentrations of radioactive materials (^238^U, ^232^Th, and ^40^K)^13^. The analysis of the radiation’s kinetic energy released per unit mass (KERMA) above the pads indicated that pad number 1 has the lowest dose rate and therefore, would serve as a background level. In contrast, the radiation field above pad number 5 is the highest, as published by the Forensic and Scientific Services. These pads are useful for conducting low dose rate calibration. Pad 5 had the highest predicted air kerma of 316 nGy/h at 0.15 m above the surface (Table 1); therefore, the RadioactivityCounter app was tested at this pad. A stand was used to adjust the distance to 0.15 m above the pad’s surface to match the published setup used to achieve this dose rate. Additional measurements were taken with the stand removed. Further measurements were acquired at the ‘background’ pad, which had the lowest dose rate. The acquisition time for determining the measurement was five minutes.

**Table 1:** Error percentages from the dose rates at the calibrated source measurements.

Average measured dose rate (µSv/h)	References dose rate (µSv/h)	% Error
105.5	104	1.44
39.58	37.3	6.11
18.56	16.6	11.8
13.25	9.3	42.47
1.25	6	− 79.16
1.83	4.1	− 55.365
1.79	3	− 40.33
2.37	2.3	3.043
1.79	1.5	19.33

#### Sunlight test

All pad’s measurements were taken in the shade. However, to determine the effect that sunlight’s measurements have, the phone was placed directly at sunlight over Pad 1 at 0.15 cm above the pad’s 1 surface. Pad 1 was chosen as it has the lowest activity. The measurements were taken for five minutes. The phone was warm to the touch after these measurements.

#### Error analysis

For error analysis, the percentage error provides the ratio of the measurement value obtained to the expected reference values. To identify percentage error, the following formula can be used:

$$ \% ~Error = \frac{{~Measured~\;dose - Reference\;~dose}}{{Reference\;dose}} \times 100. $$

The error percentage was calculated and is listed in Table 2.

**Table 2:** Radiation fields above the pads (KERMA) as predicted using ICRU data modified by Malins et al.^23^, and as measured following the RSS-131ER HPIC protocol.

Pad	Air Kerma (nGy/h at 0.15 m)	
	Predicted (ICRU53 modified by Malins et al.^23^	Measured ± 2u
1	6	9 ± 5
2	27	29 ± 7
3	65	68 ± 6
4	187	190 ± 7
5	316	312 ± 10

**Figure 3:**
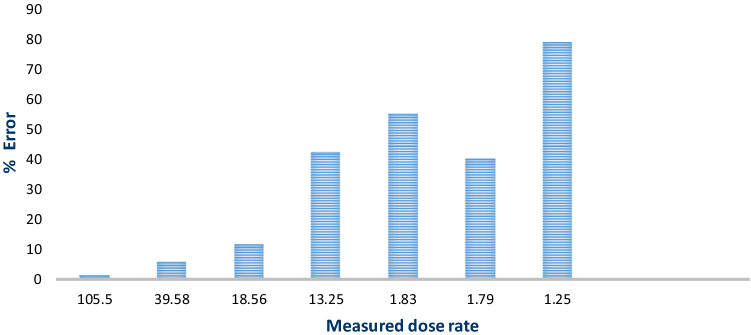
% Error percentage versus measured dose rate (µSv/h).

Percentage error was fairly low (1.44%) at high doses above 100 µSv/h, while it was very high (79.16%) at low doses of around 1.25 µSv/h. This error analysis quantitatively shows that the RadioactivityCounter app can function effectively as a radiation detector at high radiation doses; however, at low doses the app returns a high percentage of error as be presented in Fig. 3.

## Results and discussion

The RadioactivityCounter app installed on an iPhone 6s was tested. Measurements were performed with a calibrated source (^137^Cs) using the phone’s front camera. The other measurements using the back camera were performed through the use of environmental pads. The resulting data were graphically presented and analyzed using Microsoft Excel. The RadioactivityCounter app was also tested at normal background radiation levels. The indicated dose rate stabilized after 4 min at around 0.10 µSv/h.

To evaluate the minimum exposure time required for a stable signal, the iPhone 6s was irradiated for 3 min in measurement setups. From the data recorded by the app using the calibrated source (^137^Cs), the average dose rates obtained after 3 min slightly differed from the expected values, but are still somewhat acceptable. This contrasts with a previous study that found that the minimum time required for a stable signal should be 10 min or more^12^. The suitable time will differ based on the application used, the dose rate, and the type of smartphone.

### Dose rate measurements

**Figure 4:**
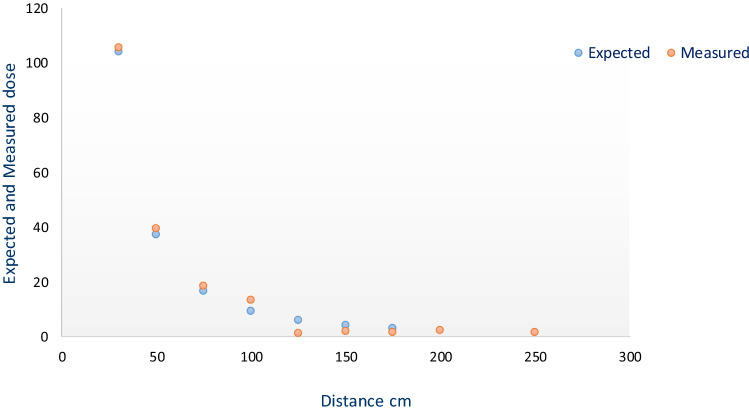
Expected and measured dose rates (µSv/h) versus distances (cm). The expected doses represent calibrated source (^137^CS) values, whereas the measured doses represent the values recorded by the RadioactivityCounter app.

**Figure 5:**
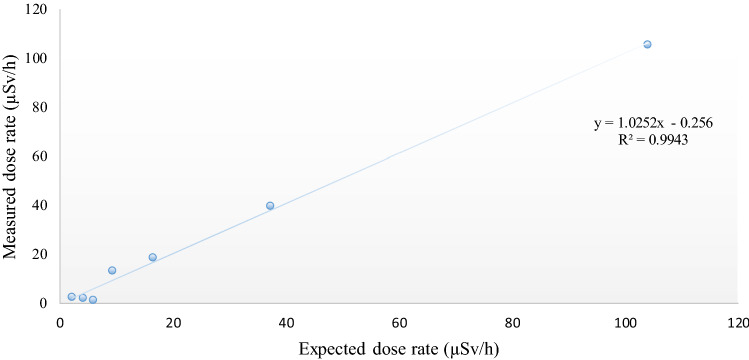
Measured and expected dose rates (µSv/h).

Radiation dose rates were measured by the phone as a function of the distance between the source and smartphone sensor. Besides, the expected dose rates determined from the calibration data related to the ARPANSA calibration of the source are shown and compared with the measured dose rates (Fig. 4). At the doses higher than approximately 20 µSv/h, the measured values tended to match the expected dose rates; however, below this level, the phone showed some variance. Furthermore, as distance increased, the dose rate decreased, and consequently, the radiation detected by the app declined. Figure 5 plots the count per minute versus the measured dose rate acquired by the RadioactivityCounter app. An approximately linear response was apparent, while at lower doses, this relationship tended to be less evident. At low dose rates of nearby 10 µSv/h, the phone’s response appeared weak. Since a dosimeter’s response to ionizing radiation ideally should not depend on the dose rate, the app was irradiated using a calibrated radioactive source (^137^Cs), with different dose rates ranging from 104 to 1.0 µSv/h and at different distances as shown in Fig. 4. The relationship between expected and measured dose rates (µSv/h) versus distance (cm) is graphically presented (Fig. 4).

The expected dose represents the values obtained from the calibrated source (Table 1) (^137^Cs-661.6 keV gamma emitter) performed at the calibration center. The measured dose represents the values obtained by the RadioactivityCounter app. The resultant data at dose rates above 20 µSv/h corresponded approximately to the expected values, as presented in Fig. 4. The measured and reference dose rates show some deviation at lower dose rates, probably due to the reduced sensitivity of CMOS sensors to low dose rates values. Figure 5 plots the measured doses versus the expected doses. An approximate linear response was observed, although, at lower dose rates around 10 µSv/h, this relationship was less clear. Therefore, the phone’s response seemed to be weaker at low dose rates.

**Figure 6:**
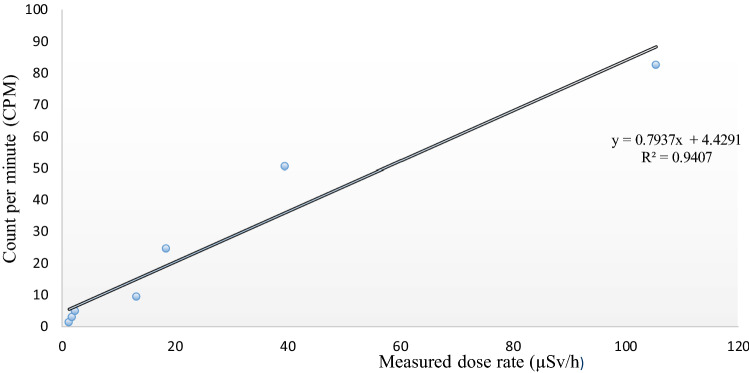
Relationship between count per minute (CPM) and measured dose rate (µSv/h).

The counts per minute recorded by the app exposed to the radioactive source (^137^Cs) were directly proportional to the dose rate above 20 µSv/h, as shown in Fig. 6. This finding contrasts with a previous study in which physicists tested an iPhone 4S at the Australian Nuclear Science and Technology Organisation (ANSTO). The recorded counts per minute were directly proportional to dose rates higher than 30 µSv/h^8^. The relatively poor performance of the RadioactivityCounter app at dose rates is lower than 10 µSv/h is demonstrated in Figs. 5 and 6, which correlates with previously published results^11^. This low sensitivity may be due to the small size of the CMOS sensor.

### Angular dependence measurements

**Figure 7:**
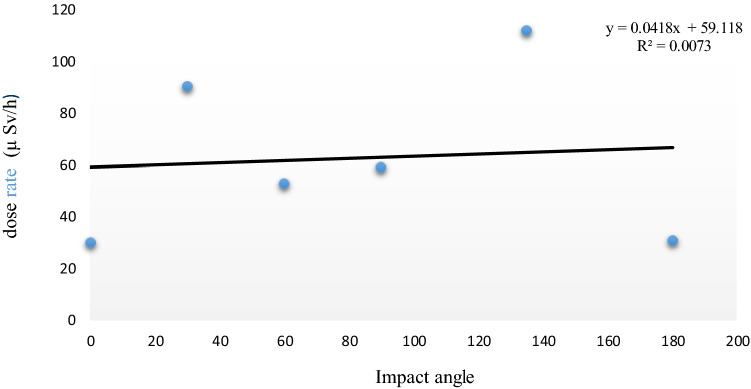
Dose rate (µSv/h) recorded by the RadioactivityCounter app versus incidence angle (degree).

The angular dependence of the measurements is shown in Fig. 7. The dots represent the dose rates at different orientation angles measured by the app. An analysis of the plots below indicates a lower dose–response at 0° and 180°. The highest responses appeared to occur at 30° and 135°, with medium responses at other angles. This suggests a definite trend concerning measurement angles. The angles of 0° and 180° are less efficient, and the radiation is less likely to interact when the pixel array is ‘flat’ concerning the beam angle.

**Figure 8:**
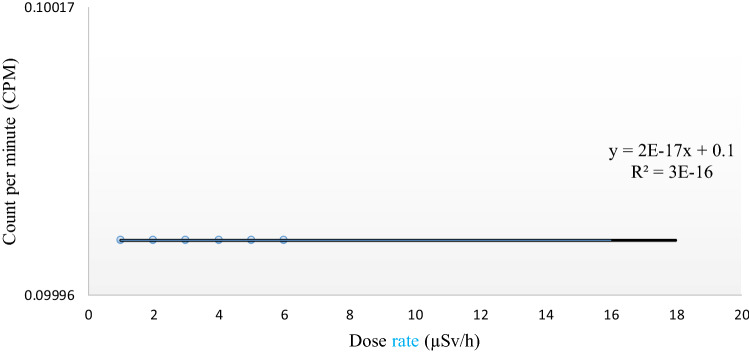
Count per minute (CPM) versus dose rate (µSv/h) measured by the RadioactivityCounter App at Pad 5.

The response of an ionizing radiation detector should also not depend on the impact angle of the radiation. Therefore, the angular dependency of the RadioactivityCounter app was evaluated. The phone’s angular response from 0° to 180° for the dose rate of 37.5 µSv/h is illustrated in Fig. 7, and this result shows that the phone’s response to radiation has an angular dependence on the orientation of the phone. The 0° and 180° angles correspond to facing the source and facing away from the source of radiation. The data in Fig. 7 shows that the phone’s front camera CMOS can detect harmful ionizing radiation at any incidental radiation angle. This helps determine the direction of the ionizing radiation from the source to the smartphone^14^. Altering the angle of the incoming gamma rays changes the shielding the gamma rays travel through to reach the sensor. For example, at certain angles, gamma rays will travel through the battery before reaching the camera. This material attenuates the gamma rays and explains why the dose rate is different at different angles. Interactions with intervening material can also generate high-energy electrons that are then detected by CMOS sensors^15^. ANSTO found that the iPhone 4 s has an angular response independent of angular impact at 50 and 150 µSv/h^8^. Other previous research found that the iPhone 4s′ angular response is independent at 1000 µSv/h^12^. In contrast, from the results shown in Fig. 8, the iPhone 6s′ angular response is dependent on its orientation at lower dose rates of around 37.5 µSv/h.

### Concrete pad measurements

The measured dose rates versus the CPM as acquired by the RadioactivityCounter app above the calibration pad number 5 (Fig. 8). No detection was recorded by the phone, as the radiation emitted from this pad is low at nearly 0.35 µSv/h^13^. This level of radiation is under the determined detectable limit.

The data recorded by the RadioactivityCounter app at pad number 5 is graphically presented as a relationship between counts per minute versus dose rate (µSv/h), as shown in Fig. 8. Since the app’s sensitivity for radiation detection is limited to 10 µSv/h, there was no detection where the radiation emitted from Pad 5 was at 0.35 µSv/h. This finding corresponds to the developer, in which the sensitivity of this app begins at 10 µSv/h^11^. The lower limit of the CMOS sensor is probably due to the CMOS sensor’s resolution and small size.

CMOS sensors have been discussed as a useful tool for dose alarms in environments where workers may be exposed to radiation^16^. However, they are not widely used in safety applications because of their low signal-to-noise ratios and limited dynamic range^16^. Nevertheless, CMOS sensors are cheap and offer accessible power. Further development of CMOS technology is expected to reduce the noise made by these sensor arrays. It is imperative to note that image sensors in commercially available cameras must be evaluated carefully before they can be used as radiation alarms^7^. To use smartphones as radiation alarms, the radiation responses of smartphones should be characterized carefully. Further research should be carried out to characterize radiation-induced pixel intensity on a smartphone’s CMOS sensor while distinguishing it from thermal noise.

**Figure 9:**
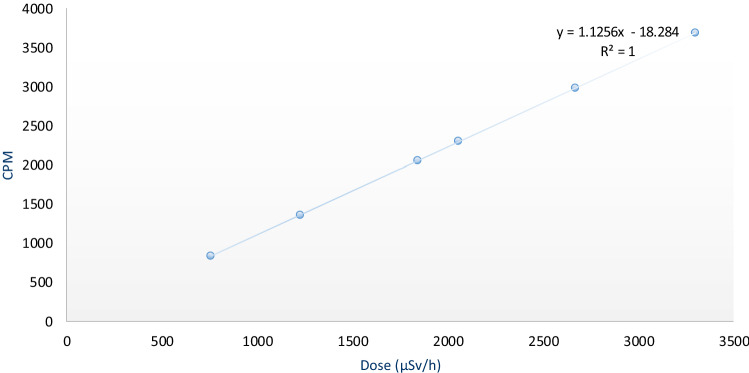
Count per minute versus dose rate (µSv/h), as recorded by the RadioactivityCounter app.

**Figure 10:**
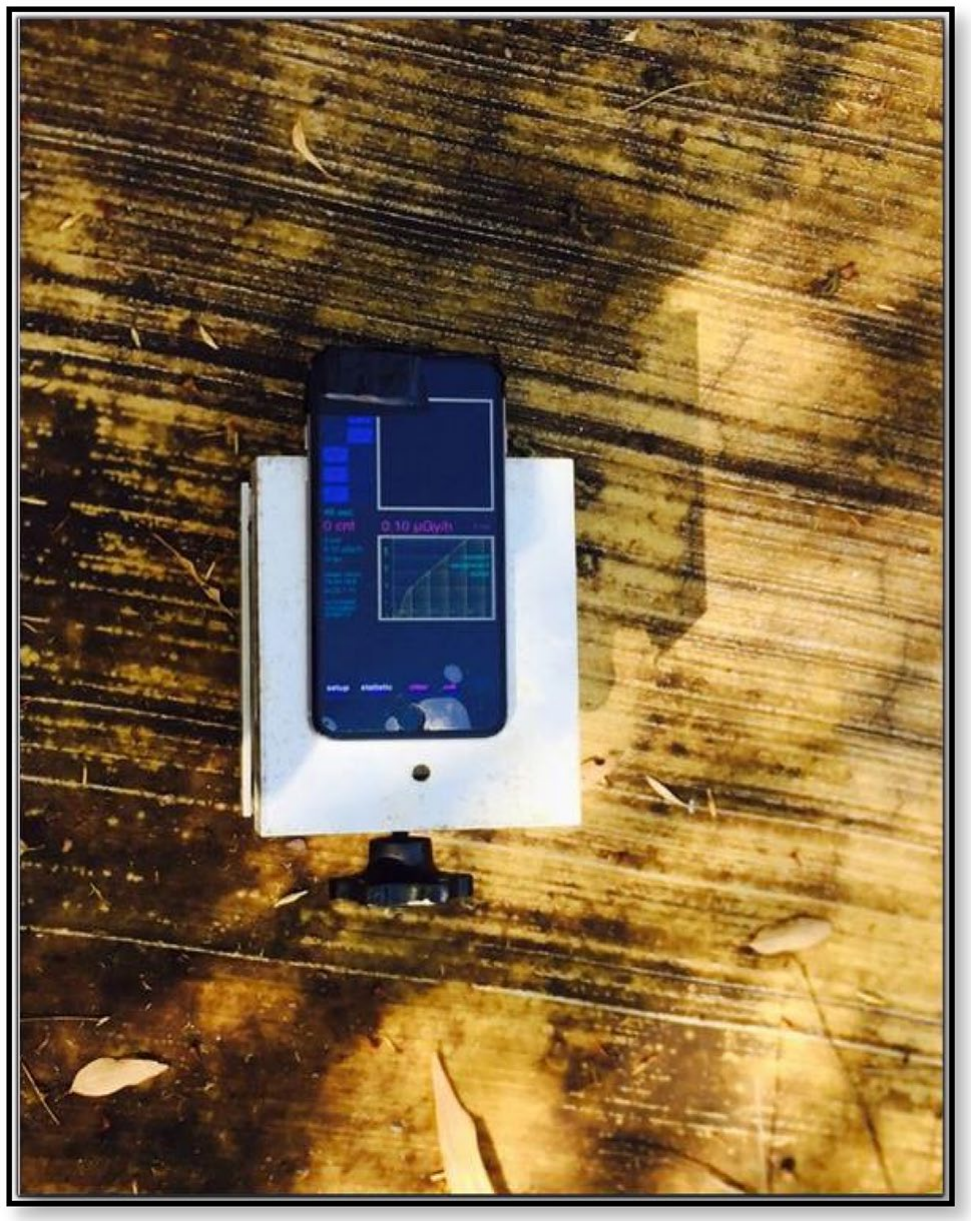
Measurement setup with stand.

**Figure 11:**
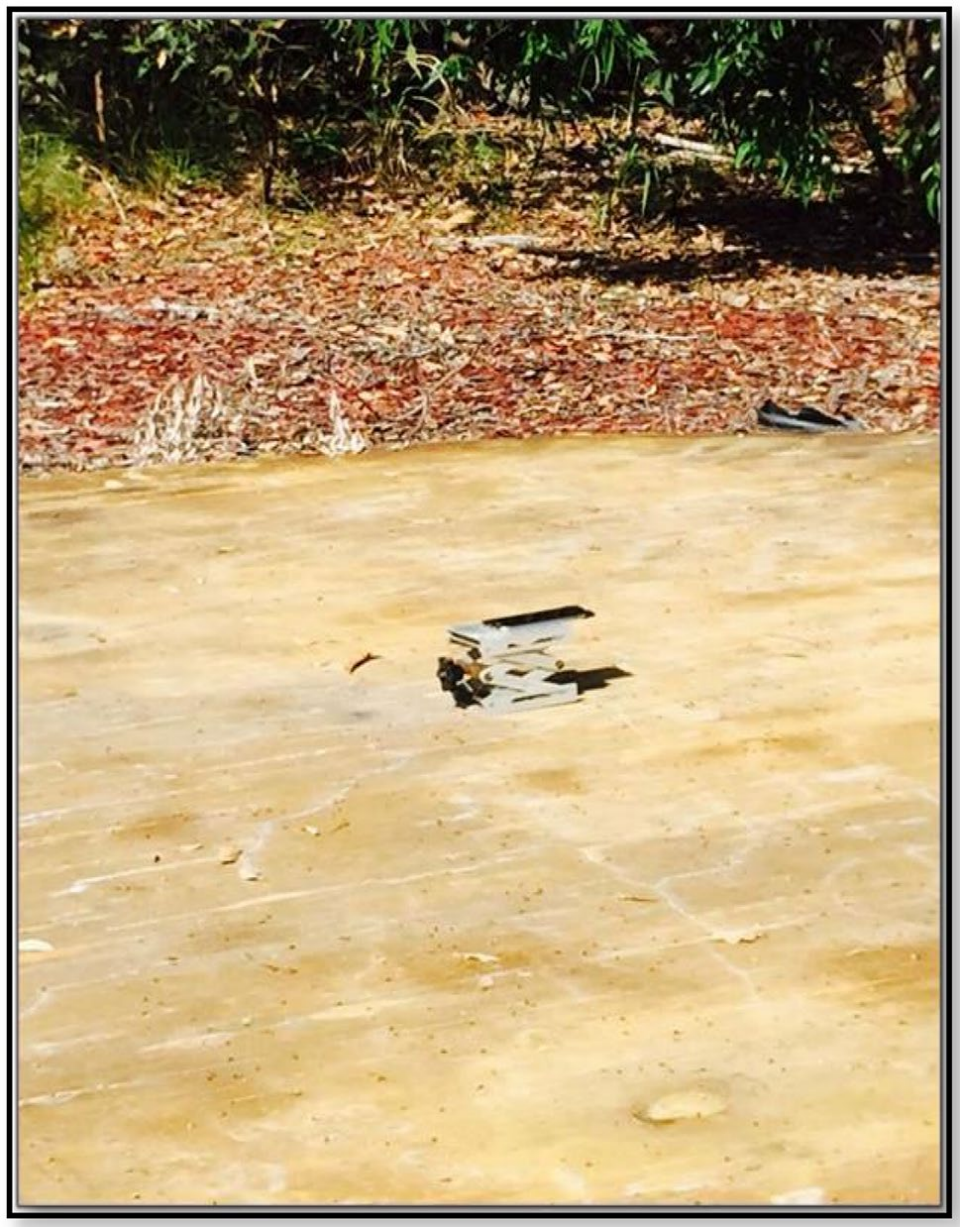
Measurement setup pointing directly at sunlight over Pad 1.

**Figure 12:**
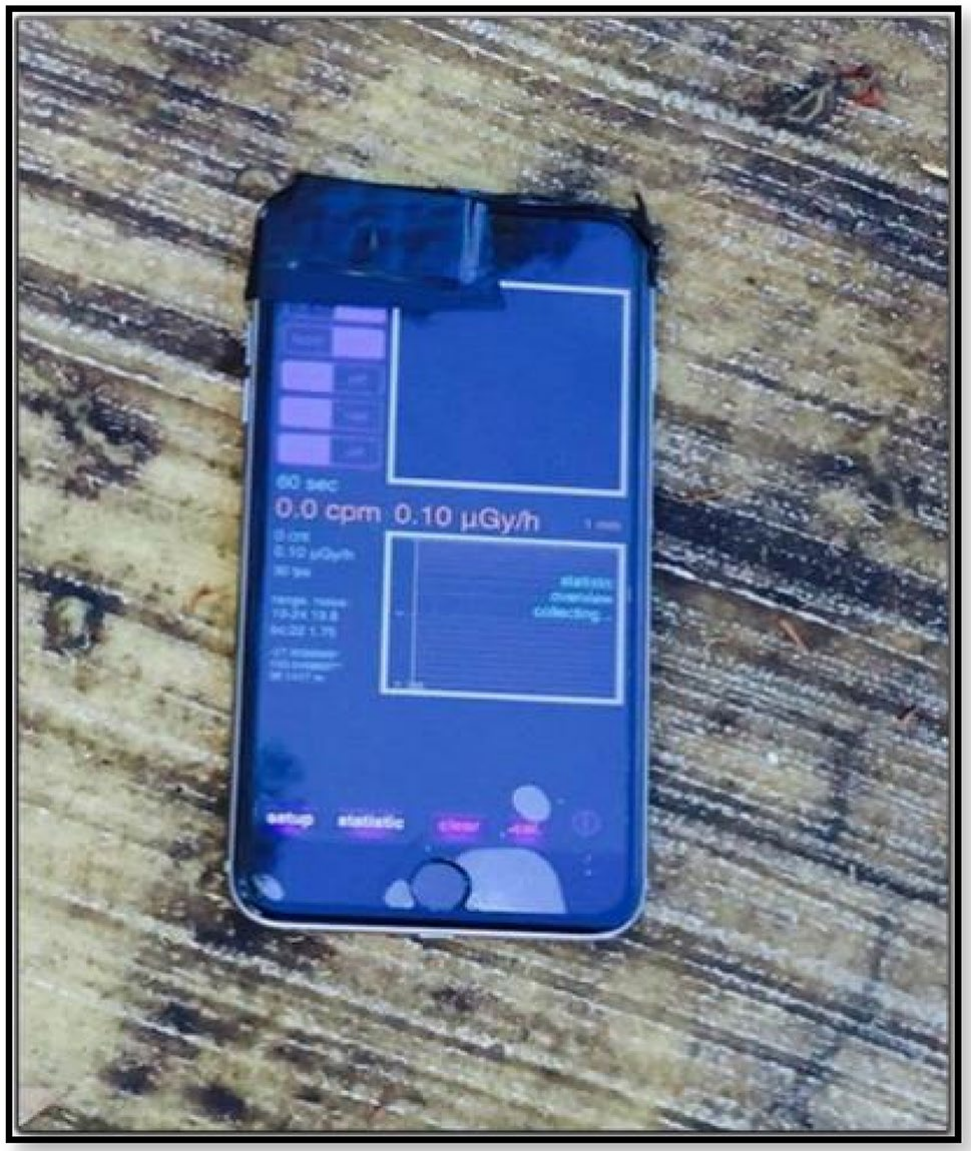
Measurement setup without stand.

Figure 9 displays the measured dose rates versus the CPM with the phone pointing toward the sun. A linear relationship was observed. Although these results do not represent radiation measurements, they demonstrate that CPM and dose rate recorded by the app are directly related. Regarding the data collected from the concrete pads, at calibration pad number 1, the app recorded no radiation, as this pad’s dose is below the limit of 10 μSv/h. However, Sunlight produced a false signal by the smartphone’s CMOS sensor, as shown in Fig. 9. A linear relationship was observed between the counts per minute and dose rates (µSv/h) recorded by the RadioactivityCounter app (Fig. 9). This demonstrates that the app can potentially be developed, which would enable a smartphone to be used as a light meter for the measurement of personal exposure to the sun (Table 2, Figs. 10, 11, 12).

Smartphones can offer customizable data with user-friendly applications that can prove more useful than conventional special-purpose equipment. Sensor quality and processing power in smartphones are continually developing. Consumer demand for high-quality image sensors is high. Modern smartphone cameras have advanced features, such as accelerated camera pixel intensity, higher image quality, and more incredible rapidity. These features allow smartphones to be especially useful in their ability to detect radiation^7^. Charge-coupled device (CCD) sensors have previously been tested for radiation detection^15^. CCD sensors convert light into an electrical charge. The charge accumulated in each cell of the image sensor is carried by the CCD array over the chip and reads each array before each pixel’s value is converted in an Analog to Digital Converter (ADC). The ADC converts the charge values to a binary signal^16^.

On the other hand, CMOS devices make use of the transistors that are located in each pixel of the sensor array. These transistors amplify and transfer the accumulated charge using wires^16^. Though they are intended to detect visible light, they can also detect the higher energies of ionizing radiation^8^. CMOS sensors have been discussed as a useful tool for dose alarms in environments in which workers may be exposed to radiation^15^. However, they are not widely used in safety applications because of their low signal-to-noise ratios and limited dynamic range^15^. Nevertheless, CMOS sensors are cheap and offer accessible power. Further development of CMOS technology is expected to reduce the noise made by these sensor arrays.

Image sensors in commercially available cameras need to be evaluated carefully before being used as radiation alarms^7^. Further research should be carried out to characterize radiation-induced pixel intensity on a smartphone’s CMOS sensor while distinguishing it from thermal noise. Several paid and free apps for radiation detection are available for smartphone devices. These apps include GammaPix, Cell Rad, RadioactivityCounter, WiFi Radiation Meter download, Pocket Geiger, EMF scanner, and others^16^.

Previous studies used old types of smartphones with limited tested parameters. It was reported that the sensitivity of the CMOS sensor considerably between different smartphone types and models due to differences in the manufacturers, production process, filters used, and variation in the gain of the CMOS amplifiers^17^. The other smartphones exhibit different CMOS sensitivity for different systems of the same type^12^. Alessandri^18^ assessed the use of three smartphones (Samsung S4, Samsung S7, and Samsung A3) for radiation detection with different radiation sources (Na-22, Zn-65, and Cs-137). The study reported that the smartphones responded contrarily to radiation. Smartphones with advanced sensors exhibited higher noise values. Jochen et al.^19^ said smartphone use for radiation dose measurements and testing different parameters for education purposes.

However, despite the low sensitivity of smartphones for the low level of radiation, they can be very useful in accident scenarios. The public may be exposed to higher dose levels^20–^^22^. Therefore, the scientific community needs to invest and refine the public’s current methods. This helps develop a well-informed society, and any user could monitor radiation to quantify the radioactivity intensity. Conventional dosimetry techniques necessitate expensive equipment with unique skills.

### Study limitations

One of the notable limitations of this study is that a suitable measurement time is required for a stable measurement, and this could be anywhere between four and ten minutes. Therefore, rapid radiation surveys that are needed (often in seconds) for rapid safety responses cannot be performed by RadioactivityCounter in which after the proper stabilization of four to ten minutes, at least one extra minute is needed to obtain a measurement. Furthermore, if the black tape is not applied correctly to the camera lens, visible rays can be detected, which invalidate the measurement. The precision of the RadioactivityCounter app may also be affected by heat or low battery. The sensitivity of the CMOS is limited to 10 µGy/h; thus, low dose rates cannot be detected. Furthermore, processing the data in the app rapidly depletes the smartphone’s battery, and black tape can damage a phone’s lens with frequent use. Alpha radiation cannot be detected as it is blocked by the housing, lens, and cover of the mobile phone. Gamma rays, X-rays, and beta particles with high energy, however, can be measured by this app.

## Conclusions

In this work, the RadioactivityCounter smartphone application was evaluated for its ability to measuring ionizing radiation. The smartphone was irradiated with a calibrated 137Cs radioactive source, calibrated concrete pads with various known concentrations of radioactive elements, and direct Sunlight. The minimum exposure time for dose–response, as well as the responses’ linearity and angular dependence, were measured. CCDs are expensive and not widely available. They can be irregularly replaced by the use of a smartphone app in detecting harmful radiation such as that caused by gamma rays. An app can enable a user to determine the dose rates of ionising radiation at different distances and different directions from the ionising radiation’s source. The advantage of using a smartphone app in radiation detection is that it is cheap, simple to operate and accessible, since many people possess smartphones. In particular, the RadioactivityCounter application functions as a radiation detector at dose rates of higher than 10 µGy/h without the need for any hardware beyond a smartphone and a piece of electrical tape.

The advantage of using a smartphone app in radiation detection is that it is cheap, simple to operate, and accessible since many people possess smartphones. In particular, the RadioactivityCounter application functions as a radiation detector at dose rates of higher than 10 µGy/h without the need for any hardware beyond a smartphone and a piece of electrical tape. This study has shown that the RadioactivityCounter app is a readily available and useful tool for warning members of the public and industrial workers of hazardous doses of ionizing radiation that could be released in accidents.

## References

[1] Ribeiro ASF, Husson O, Drey N, Murray I, May KA, Thurston J, Oyen WJG (2020). Radiation exposure awareness from patients undergoing nuclear medicine diagnostic 99mTc-MDP bone scans and 2-deoxy-2- (18F) fluoro-D-glucose PET/computed tomography scans. Nucl. Med. Commun..

[2] Sanders CL (2017). Radiobiology and Radiation Hormesis: New Evidence and Its Implications for Medicine and Society.

[3] United Nations Scientific Committee on the Effects of Atomic Radiation. Sources and effects of ionizing radiation. Volume I: Sources. UNSCEAR 2008 Report to the general assembly with scientific annexes A and B. United Nations (2010).

[4] Ojovan MI, Lee WE, Kalmykov SN (2019). Background Radiation. An Introduction to Nuclear Waste Immobilisation.

[5] Drukier, G. A., Rubenstein, E. P., Solomon, P. R. , Wójtowicz, M. A. & Serio, M. A. Low cost, pervasive detection of radiation threats. In *2011 IEEE International Conference on Technologies for Homeland Security (HST)* 365–371 (2011).

[6] UNSCEAR. *Report 2008. United Nations Scientific Committee on the Effects of Atomic Radiation*. 2008 Report to the general assembly.

[7] Kang HG, Song JJ, Lee K, Nam KC, Hong SJ, Kim HC (2016). An investigation of medical radiation detection using CMOS image sensors in smartphones. Nucl. Instrum. Methods Phys. Res. Sect. A.

[8] ANSAT Organisation. *Smartphone Radiation Detector ‘App’ Tests Positive-ANSTO* (2020). http://www.ansto.gov.au/AboutANSTO/MediaCentre/News/ACS049898 (Accessed 18 August 2020).

[9] Igoe D, Parisi A, Carter B (2013). Characterization of a smartphone camera's response to ultraviolet A radiation. Photochem. Photobiol..

[10] Hartmann VH, Freudenberg R, Kotzerke J (2013). On the article Kaireit T et al. Smartphones now even smarter-possibility of using a dose warner. RoFo Fortschritte auf dem Gebiete der Rontgenstrahlen und der Nuklearmedizin.

[11] Rolf-Dieter, K. *Radio A Help. RadioactivityCounter for Mobile Phones.* http://www.hotray-info.de/html/radioahelp.html (Accessed 06 March 2018).

[12] Hoey V (2015). Radiation dosimetry properties of smartphone CMOS sensors. Radiat. Prot. Dosim..

[13] Wallace J (2016). Establishing a NORM based radiation calibration facility. J. Environ. Radioact..

[14] Siti Rozaila Z, Khandaker MU, Wahib NB, Hanif bin Abdul Jilani MK, Abdul Sani SF, Bradley DA (2020). Thermoluminescence characterization of smartphone screen for retrospective accident dosimetry. Radiat. Phys. Chem..

[15] Cogliati, J., Derr, K. W. & Wharton, J. Using CMOS sensors in a cellphone for gamma detection and classification. Preprint at http://arXiv.org/1401.0766 (2014).

[16] Nordqvist, C. *Radiation Poisoning: Sources, Effects, and Treatment* (2015). https://www.medicalnewstoday.com/articles/219615.php. Accessed October 12, 2020.

[17] Burggraaff O (2019). Standardized spectral and radiometric calibration of consumer cameras. Opt. Express.

[18] Alessandri S (2017). In the field feasibility of a simple method to check for radioactivity in commodities and in the environment. PLoS Curr..

[19] Jochen K (2014). iRadioactivity—Possibilities and limitations for using smartphones and tablet PCs as radioactive counters: Examples for studying different radioactive principles in physics education. Phys. Teach..

[20] Qing-Yang W (2017). Surveying ionizing radiations in real time using a smartphone. Nucl. Sci. Technol..

[21] Goh, V, *et al.* An alternative approach to background radiation monitoring using smartphone-coupled personal dosimeter POLISMART in Shimokita Peninsula, Japan. *Radiat. Environ. Med*. **9**(2), 84–92 (2020).

[22] EJP-CONCERT, European Joint Programme for the Integration of Radiation Protection Research, H2020- 662287. D9.133 – Review of applications and devices for citizen dose measurement. Available at: https://territories.eu/assets/files/publications/D9.67_Stakeholders-panel-results_approved03072019.pdf (2019). Accessed April 2, 2021.

[23] Malins, A, Machida, M, Saito, K. Comment on Update of 40K and 226Ra and 232Th series gamma-to-dose conversion factors for soil. *J. Environ.Radioact*. **144**, 179–180 (2015). 10.1016/j.jenvrad.2015.01.009 25698415

